# CRISPR and yeast two-hybrid screens identify CD81 as a proviral host factor for PRRSV

**DOI:** 10.1128/jvi.01033-26

**Published:** 2026-09-14

**Authors:** Yongjie Chen, Zhan He, Yanfei Pan, Yunyan Luo, Baoying Huang, Yi Zhang, Guohao Li, Chunhe Guo

**Affiliations:** 1 Guangdong Laboratory for Lingnan Modern Agriculture, State Key Laboratory of Animal Disease Control and Prevention, Key Laboratory of Zoonosis Prevention and Control of Guangdong Province, College of Veterinary Medicine, South China Agricultural University 12526, Guangzhou, Guangdong, People's Republic of China; University of Michigan Medical School, Ann Arbor, Michigan, USA

**Keywords:** CRISPR library, host-virus interaction, CD81, PRRSV GP5, autophagic degradation

## Abstract

Porcine reproductive and respiratory syndrome virus (PRRSV) remains a major economic burden on the global swine industry. Identifying host factors that mediate viral invasion is critical for developing antiviral strategies. Here, using a CRISPR screen targeting genes encoding cell surface CD proteins combined with yeast two-hybrid screening, we identified the tetraspanin CD81 as a novel host factor that promoted PRRSV infection by directly interacting with viral glycoprotein 5 (GP5). CD81 knockdown reduced PRRSV infection, whereas CD81 overexpression enhanced it. Consistently, antibody-mediated blockade of CD81 restricted infection in both Marc-145 cells and primary porcine alveolar macrophages. Mechanistic analysis showed that CD81 mainly facilitated viral internalization, and its C-terminal transmembrane region was required for GP5 binding and efficient infection. During infection, CD81 underwent autophagic degradation mediated by the selective autophagy receptor NDP52, suggesting a host restriction response that reduced CD81 availability. PRRSV countered this process by promoting NDP52 degradation, thereby stabilizing CD81 and favoring infection. These findings identify CD81 as both a key factor in PRRSV pathogenesis and a potential target for antiviral intervention.

**IMPORTANCE:**

Porcine reproductive and respiratory syndrome virus (PRRSV) remains one of the most economically important swine pathogens worldwide, yet effective antiviral strategies are lacking because of its extensive genetic diversity and immune evasion. Here, we identified the tetraspanin CD81 as a previously unrecognized host factor that promoted efficient PRRSV infection. CD81 directly interacted with the viral glycoprotein GP5 to facilitate viral internalization, whereas disruption of CD81 by genetic depletion or antibody blockade markedly restricted infection. We further demonstrated that CD81 abundance was controlled by NDP52-mediated selective autophagy as an intrinsic antiviral mechanism, which PRRSV overcame by degrading NDP52 to stabilize CD81 and enhance viral replication. These findings reveal a previously unrecognized virus-host interaction that governs PRRSV entry and identify CD81 as a promising host-directed therapeutic target.

## INTRODUCTION

Porcine reproductive and respiratory syndrome (PRRS), caused by porcine reproductive and respiratory syndrome virus (PRRSV), is a widespread and highly contagious disease of swine. It compromises herd health by causing reproductive failure in sows, respiratory disease in piglets, and increased susceptibility to secondary infections, thereby imposing substantial economic losses on the global swine industry (1, 2). PRRSV is an enveloped, positive-sense RNA virus of the family *Arteriviridae*. Its genome is approximately 15 kb in length and contains at least 11 open reading frames (ORFs) (3, 4). Based on phylogenetic classification, PRRSV is divided into two species, PRRSV-1 (*Betaarterivirus suid 1*) and PRRSV-2 (*Betaarterivirus suid 2*). Although these two species share only approximately 60% sequence identity, they cause broadly similar clinical manifestations in infected pigs (5–8). ORF1a and ORF1b encode non-structural proteins (nsps) required for viral replication, whereas ORFs 2–7 encode structural proteins involved in virion assembly (9–11). A defining feature of PRRSV infection is its strict cellular tropism. The virus preferentially infects macrophages, particularly porcine alveolar macrophages (PAMs), which provide the main site for viral replication and support systemic dissemination (12, 13). In addition, the high rates of mutation and recombination in PRRSV accelerate viral evolution and contribute to immune evasion, making disease prevention and control particularly difficult (14, 15).

Productive viral infection begins with the attachment of virions to specific host membrane proteins, followed by entry into susceptible cells (16). In PRRSV infection, CD163 is recognized as a key receptor that supports viral uncoating and subsequent release of the viral genome into the cytoplasm (17). The cluster of differentiation (CD) proteins comprise a group of cell-surface molecules whose expression in leukocyte lineages changes with cellular differentiation, maturation, and activation (18). Many CD proteins function as receptors or ligands and are involved in processes, such as immune activation, cell growth, and development. Among them, CD81 is a widely expressed tetraspanin containing four transmembrane domains (TM1–TM4), a small extracellular loop (SEL), a large extracellular loop (LEL), and short cytoplasmic tails (19, 20). As a membrane-organizing scaffold, CD81 participates in the formation of membrane microdomains and regulates protein trafficking, cell fusion, and signal transduction (21). Several pathogens have been shown to exploit CD81 during infection (22, 23). For example, the LEL of human CD81 serves as an essential entry factor for hepatitis C virus (HCV) through its interaction with the viral E2 glycoprotein (24). CD81 has also been implicated in the infection cycles of other viruses, including chikungunya virus (CHIKV) (25), equine arteritis virus (EAV) (26), herpes simplex virus 1 (HSV-1) (27), and porcine circovirus type 2 (PCV2) (28). Despite its established role as a viral co-receptor, whether and how CD81 participates in PRRSV infection remained unknown.

In this study, we validated the proviral role of CD81 in PRRSV infection through CRISPR and yeast two-hybrid (Y2H) screens, which demonstrated that its C-terminal transmembrane domain is crucial for both major viral glycoprotein 5 (GP5) binding and viral infection. We further found that the host mounts an autophagy-mediated defense by targeting CD81 for degradation via the selective autophagy receptor NDP52. PRRSV, however, subverts this defense by degrading NDP52 through the same autophagic pathway, thereby stabilizing CD81 and facilitating infection. These findings define CD81 as an entry-associated host factor for PRRSV and uncover a reciprocal host-virus regulatory axis involving CD81 and selective autophagy, providing a mechanistic basis for targeting CD81 in antiviral development.

## RESULTS

### CRISPR and Y2H screens identify CD81 as a crucial host factor for PRRSV infection

**Fig 1:**
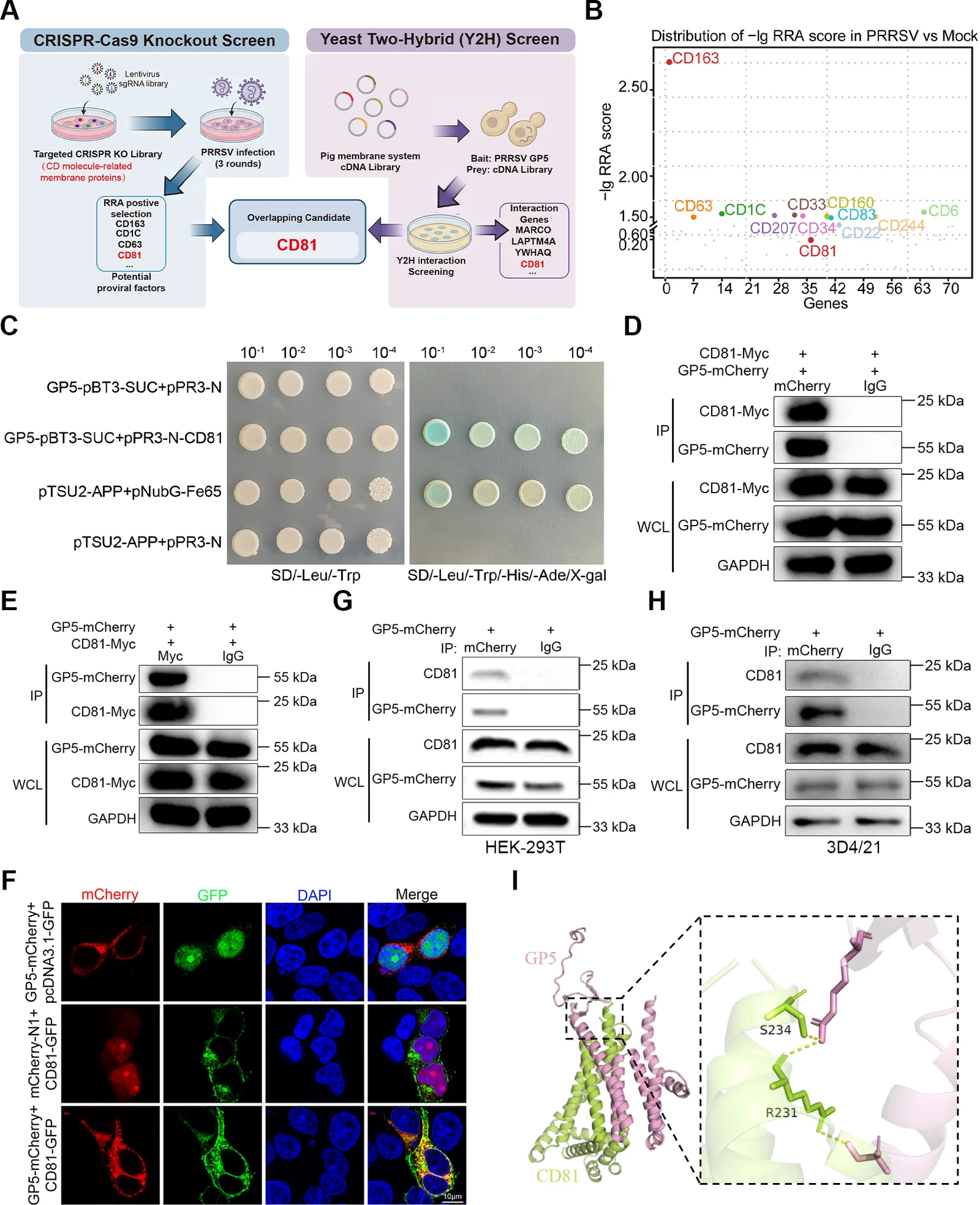
Identification of CD81 as a crucial host factor for PRRSV infection via CRISPR and Y2H screens. (**A**) Schematic diagram of CRISPR and Y2H screens. (**B**) The screening was performed in PRRSV-infected Marc-145-Cas9 cells, with gene enrichment ranked according to the highest robust rank aggregation (RRA) scores. (**C**) Yeast strain NMY51 was co-transformed with bait plasmid GP5-pBT3-SUC and prey plasmid pPR3-N-CD81 to study protein-protein interactions. Positive (pTSU2-APP/pNubG-Fe65) and negative (GP5-pBT3-SUC/pPR3-N) control transformations were included. Transformants were selected on synthetic dropout (SD) medium. Robust colony growth on stringent quadruple-dropout medium (SD/-Leu/-Trp/-His/-Ade) demonstrated GP5-CD81 interaction, while viability was confirmed on double-dropout medium (SD/-Leu/-Trp). (**D and E**) HEK-293T cells were transfected with CD81-Myc and GP5-mCherry. Co-IP was performed, followed by western blot. (**F**) HEK-293T cells were transfected with GP5-mCherry (or empty vector (EV)) and CD81-GFP (or EV) to assess colocalization by confocal microscopy. (**G and H**) HEK-293T and 3D4/21 cells were transfected with GP5-mCherry for subsequent Co-IP analysis. (**I**) The CD81-GP5 interaction was predicted using AlphaFold3.

The host protein CD163 serves as the primary and essential receptor for PRRSV infection, and CD163-knockout pigs are completely resistant to the virus, demonstrating that CD163 functions as a proviral factor (29, 30). To investigate whether other CD proteins are involved in this process, we designed a custom sgRNA library targeting this family and performed a positive-selection CRISPR screen in PRRSV-infected cells (Fig. 1A). As expected, CD163 emerged as the top hit, validating the robustness of our screening system (Fig. 1B and File S1). In addition, several other CD-family proteins, such as CD81 and CD63, were enriched in the screen (Fig. 1B). Concurrently, given the central role of the major envelope glycoprotein GP5 in viral entry, we performed a Y2H screen using GP5 as bait to identify its interacting host factors. This screen not only confirmed the known GP5-MARCO interaction (9) but also identified CD81 as a novel binding partner (Fig. 1A and C and File S2). This dual identification prompted us to further investigate the functional role of CD81 in PRRSV infection.

To validate this interaction in mammalian cells, HEK-293T cells were co-transfected with mCherry-tagged GP5 and Myc-tagged CD81 for 24 h. Co-immunoprecipitation (Co-IP) using anti-mCherry or anti-Myc antibodies confirmed a GP5-CD81 interaction (Fig. 1D and E). Consistent with this, confocal microscopy revealed strong colocalization of CD81 and GP5 (Fig. 1F), and Co-IP further demonstrated that GP5 binds endogenous CD81 (Fig. 1G and H). Finally, a structural model predicted by AlphaFold3 supported the feasibility of a direct CD81-GP5 interaction (Fig. 1I). Collectively, these findings indicate that CD81 likely serves an important function during PRRSV infection.

### Identification of the critical domain in the interaction between GP5 and CD81

**Fig 2:**
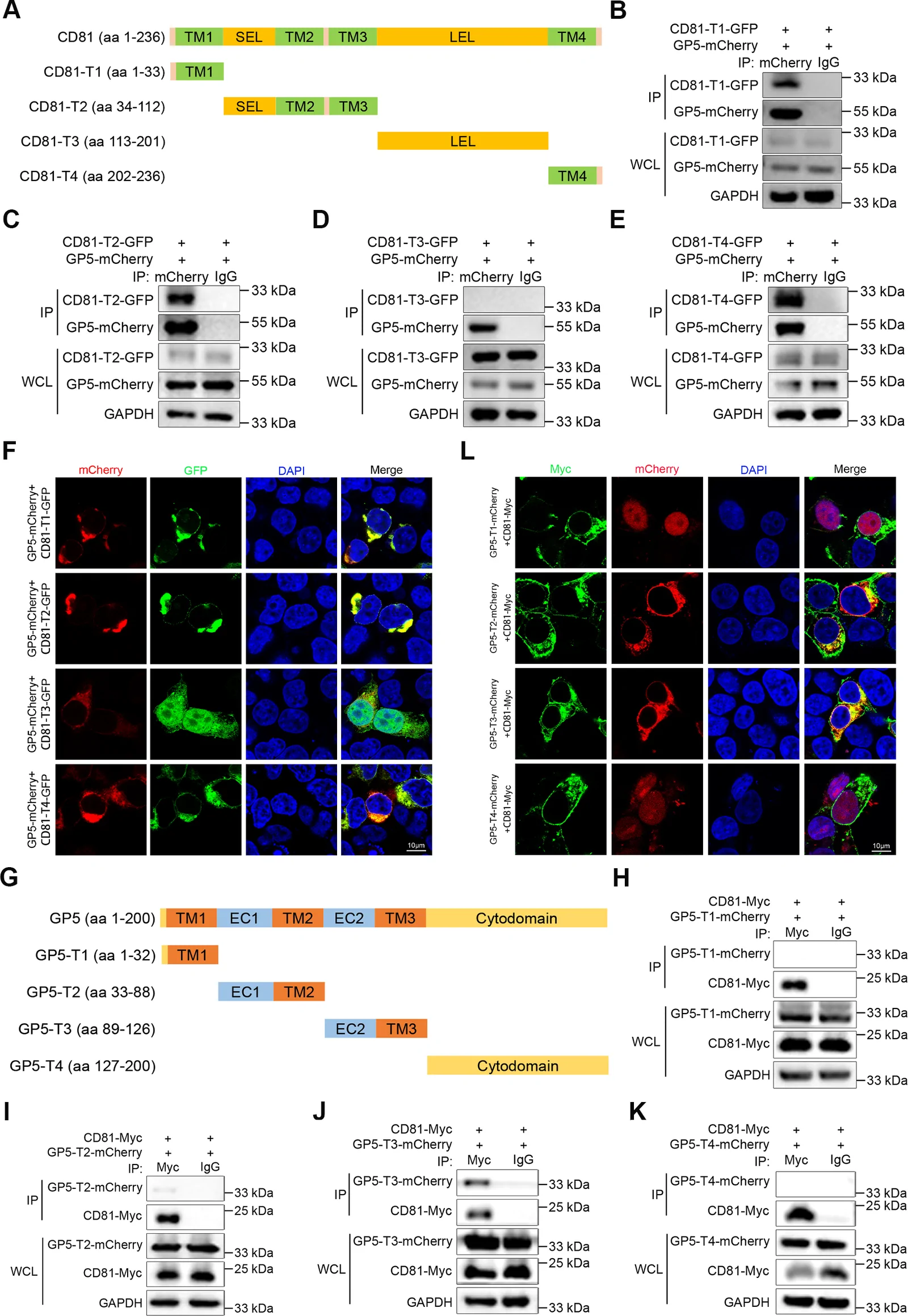
Identification of the critical domain mediating the GP5-CD81 interaction. (**A**) Schematic diagram of the CD81 truncated mutants. (**B–E**) HEK-293T cells were transfected with GP5-mCherry and the CD81 truncations, followed by Co-IP analysis. (**F**) HEK-293T cells were transfected with GP5-mCherry and the CD81 truncations, followed by confocal microscopy. (**G**) Schematic diagram of the GP5 truncated mutants. (**H–K**) HEK-293T cells were transfected with CD81-Myc and the GP5 truncations, followed by Co-IP analysis. (**L**) HEK-293T cells were transfected with CD81-Myc and the GP5 truncations, followed by confocal microscopy.

Based on the domain architecture of CD81, a series of GFP-tagged truncation mutants spanning residues 1–33, 34–112, 113–201, and 202–236 were generated (Fig. 2A). Co-IP analysis showed that truncations containing transmembrane domains (aa 1–33, 34–112, 202–236) interacted with GP5, whereas the large extracellular loop (LEL; aa 113–201) did not (Fig. 2B through E). Confocal microscopy revealed that GP5 colocalized with the transmembrane-containing truncations in the cytoplasm, while the LEL domain localized predominantly to the nucleus and failed to colocalize with GP5 (Fig. 2F). These results demonstrate that the subcellular localization of CD81 domains critically determines their interaction with GP5.

To delineate the CD81-binding region of GP5, we generated a series of mCherry-tagged GP5 truncations based on its predicted domain architecture (31) (Fig. 2G). Co-IP identified the two ectodomains (aa 33–88 and 89–126) as necessary and sufficient for interaction with CD81 (Fig. 2I and J), while the N-terminal signal peptide (aa 1–32) and C-terminal cytoplasmic domain (aa 127–200) showed no binding (Fig. 2H and K). Subcellular localization analysis showed that CD81 was mainly colocalized in the cytoplasm with GP5 fragments containing ectodomain regions. By contrast, the non-interacting N- and C-terminal GP5 fragments were predominantly distributed in the nucleus and showed limited overlap with CD81 (Fig. 2L). These results indicate that the physical association between CD81 and GP5 is closely related to their cytoplasmic colocalization, in which the CD81 transmembrane regions and GP5 ectodomain-containing fragments are likely to provide the accessible structural basis for their interaction.

### CD81 acts as a proviral host factor for PRRSV

**Fig 3:**
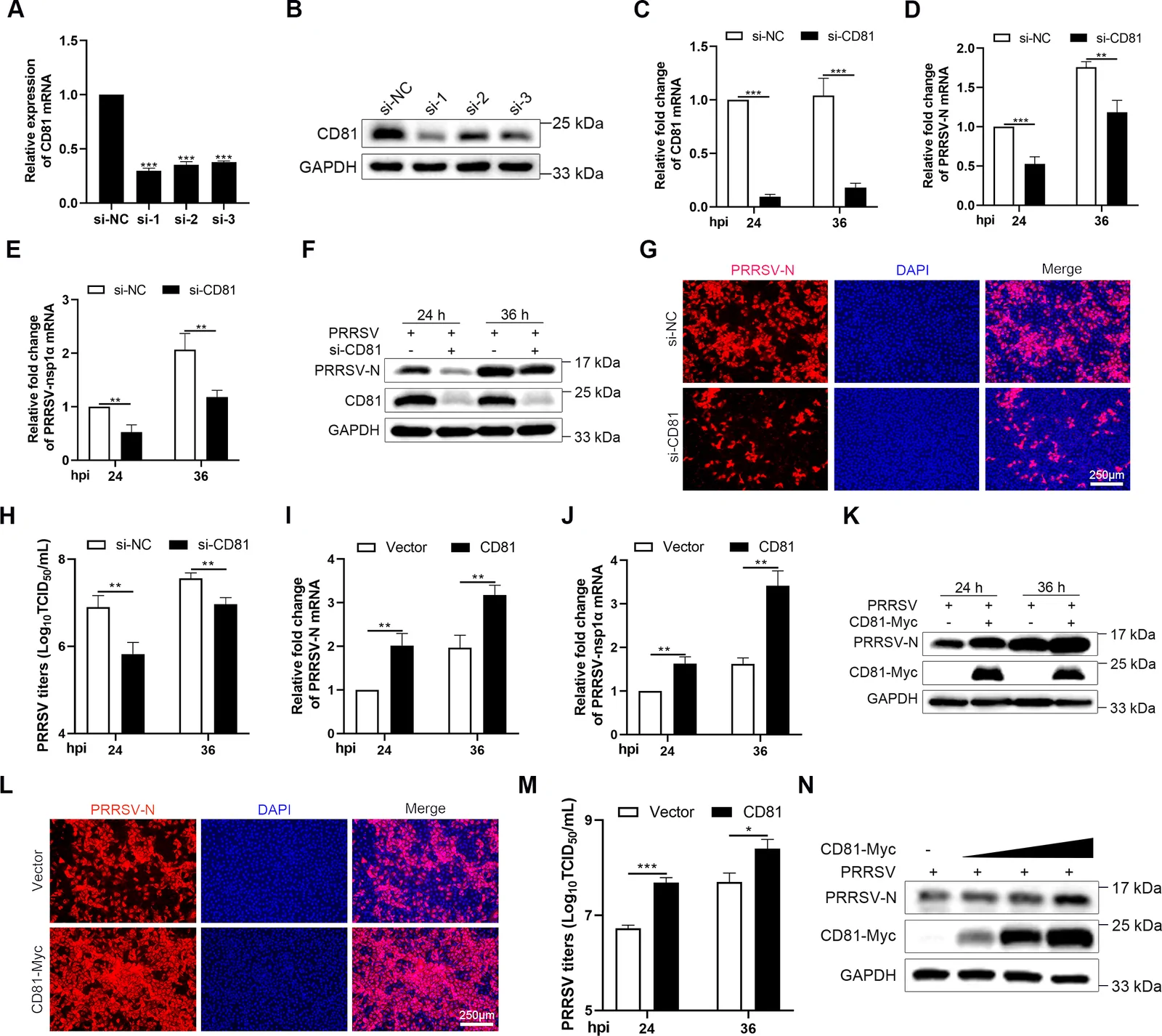
CD81 is a proviral host factor for PRRSV. (**A and B**) Marc-145 cells were transfected with CD81-specific siRNAs for 24 h and then analyzed by RT-qPCR and western blot. (**C–F**) Marc-145 cells were transfected with CD81 siRNA-1 for 24 h and infected with PRRSV CH-1a (MOI 0.5) for the indicated times, followed by RT-qPCR and western blot. (**G**) Marc-145 cells were transfected with CD81 siRNA-1 for 24 h and infected with PRRSV CH-1a (MOI 0.5) for 24 h, followed by IFA. (**H**) Marc-145 cells were transfected with CD81 siRNA-1 for 24 h and infected with PRRSV CH-1a (MOI 0.5) for the indicated times, followed by TCID₅₀ assay. (**I–K**) Marc-145 cells were transfected with CD81-Myc or EV for 24 h and infected with PRRSV CH-1a (MOI 0.5) for the indicated times, followed by RT-qPCR and western blot analysis. (**L**) Marc-145 cells were transfected with CD81-Myc or EV for 24 h and infected with PRRSV CH-1a (MOI 0.5), followed by IFA at 24 h post-infection. (**M**) Marc-145 cells were transfected with CD81-Myc or EV for 24 h and infected with PRRSV CH-1a (MOI 0.5) for the indicated times, followed by TCID₅₀ assay. (**N**) Marc-145 cells were transfected with increasing amounts of CD81-Myc for 24 h and infected with PRRSV CH-1a (MOI 0.5) for 24 h, followed by western blot analysis. Data are shown as mean ± SEM (*n* = 3). Statistical significance was determined using one-way ANOVA or Student’s *t*-test as appropriate. *P < 0.05, **P < 0.01, ***P < 0.001; ns, not significant.

To examine whether endogenous CD81 contributes to PRRSV infection, specific siRNAs targeting CD81 were designed and evaluated. All three siRNAs reduced CD81 expression at both the mRNA and protein levels relative to the negative control (si-NC). Among them, si-1 produced the most efficient knockdown and was therefore used in subsequent experiments (Fig. 3A and B). Silencing CD81 markedly impaired PRRSV replication, as shown by the reduction in viral genomic RNA copies (Fig. 3C through E), decreased viral N protein expression (Fig. 3F and G), and lower infectious viral titers (Fig. 3H). Conversely, to test whether CD81 overexpression promotes infection, we transfected Marc-145 cells with a CD81-expressing plasmid prior to PRRSV challenge. CD81 overexpression significantly increased viral genomic RNA levels (Fig. 3I and J), enhanced N protein expression (Fig. 3K and L), and elevated viral titers (Fig. 3M). Furthermore, a positive correlation was observed between CD81 expression levels and the extent of viral replication promotion (Fig. 3N). Collectively, these results indicate that CD81 expression is both necessary and sufficient to promote PRRSV replication.

### CD81 is involved in PRRSV internalization

**Fig 4:**
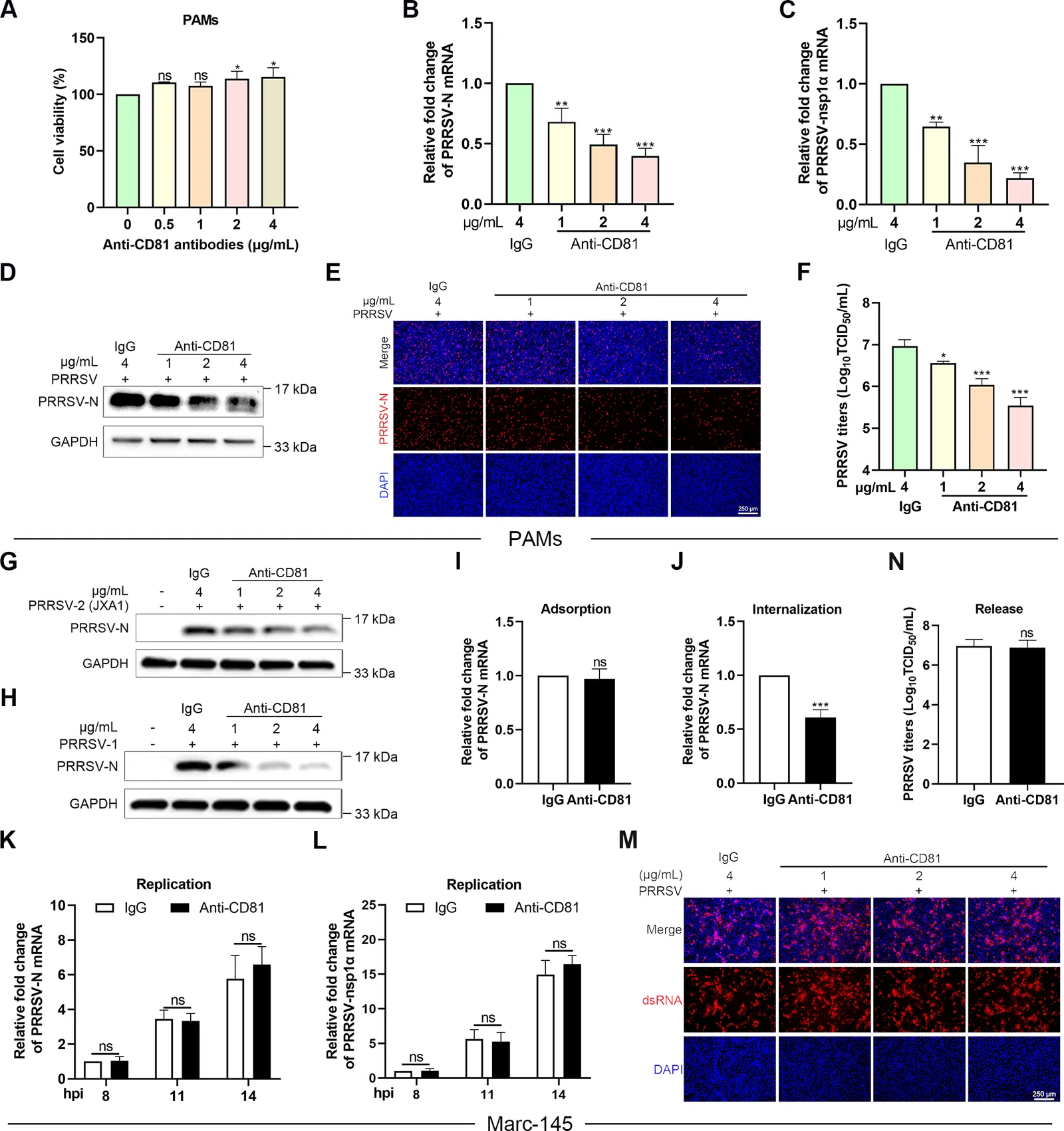
CD81 mediates PRRSV internalization**.** (**A**) PAMs were treated with varying concentrations of anti-CD81 antibody for 24 h, and cell viability was assessed using a CCK-8 assay. (**B–F**) PAMs were pre-incubated with specific antibodies or isotype-control IgG for 2 h at 37°C before infection with PRRSV strain JXA1 (MOI 0.5), with antibodies/IgG remaining during culture. At 24 h post-infection, cells were analyzed by RT-qPCR (**B and C**), western blot (**D**), IFA (**E**), and TCID₅₀ (**F**) assay. (**G and H**) Marc-145 cells were pre-incubated with specific antibodies or isotype-control IgG for 2 h at 37°C, then infected with PRRSV-2 strain JXA1 (MOI 0.5) or PRRSV-1 (MOI 0.5) for 24 h, followed by western blot. (**I**) Marc-145 cells were pre-incubated with CD81-specific antibodies or isotype-control IgG (4 μg/mL) for 2 h at 37°C, then cooled to 4°C for 1 h and infected with PRRSV CH-1a (MOI 10) at 4°C for 2 h. After washing with cold PBS, cell-associated viral RNA was quantified by RT-qPCR. (**J**) Marc-145 cells were pre-incubated with CD81-specific antibodies or isotype-control IgG (4 μg/mL) for 2 h at 37°C, infected with PRRSV CH-1a (MOI 10) at 37°C for 2 h, washed with cold PBS, and cell-associated viral RNA was quantified. (**K and L**) Marc-145 cells were infected with PRRSV CH-1a (MOI 0.5) at 37°C for 2 h, treated with CD81-specific antibodies or isotype-control IgG (4 μg/mL) for the indicated durations, and analyzed by RT-qPCR for viral N and nsp1α mRNA levels. (**M**) Marc-145 cells were infected with PRRSV CH-1a (MOI 0.5) at 37°C for 2 h, treated with CD81-specific antibodies or isotype-control IgG, and analyzed by IFA for viral dsRNA at 14 h post-infection. (**N**) Marc-145 cells were infected with PRRSV CH-1a (MOI 0.5) at 37°C for 22 h, treated with CD81-specific antibodies or isotype-control IgG (4 μg/mL) for 2 h, and viral titers in supernatants were determined by TCID₅₀ assay. Data are shown as mean ± SEM (*n* = 3). Statistical significance was determined using one-way ANOVA or Student’s *t*-test as appropriate. *P < 0.05, **P < 0.01, ***P < 0.001; ns, not significant.

Given that CD81 is a host membrane protein, we asked whether blocking it with a specific antibody could inhibit PRRSV infection *in vitro*. First, a CCK-8 assay confirmed that the anti-CD81 or anti-IgG antibodies (up to 4 μg/mL) exhibited no cytotoxicity in Marc-145 cells (Fig. S1A). Pretreating cells with the antibody for 2 h prior to infection resulted in a dose-dependent inhibition of PRRSV replication, as shown by decreased levels of viral N and nsp1α mRNA (Fig. S1B and C) and protein (Fig. S1D and E), along with reduced viral titers (Fig. S1F). We next evaluated the antiviral effect in PAMs, the natural target cells of PRRSV. The antibody showed no cytotoxicity in PAMs (Fig. 4A). Pretreatment with the antibody prior to infection similarly led to a dose-dependent reduction in viral mRNA (Fig. 4B and C), N protein expression (Fig. 4D and E), and progeny virus production (Fig. 4F). The antibody also effectively suppressed infection by other PRRSV strains, including the highly pathogenic PRRSV-2 JXA1 and PRRSV-1 (Fig. 4G and H). These results show that antibody-mediated blockade of CD81 restricts PRRSV infection in both cell line and primary cell models, supporting the possibility that CD81 may serve as a host-directed antiviral target.

To define the stage at which CD81 contributes to the PRRSV life cycle, we examined viral attachment, entry, replication, and release separately. Blocking CD81 did not alter viral attachment to host cells (Fig. 4I), whereas treatment with an anti-CD81 antibody significantly reduced viral internalization (Fig. 4J). After viral entry, antibody treatment had no detectable effect on the RNA levels of viral N or nsp1α during replication (Fig. 4K and L). Consistently, dsRNA staining indicated that viral replication complexes were still formed under CD81 blockade (Fig. 4M). Viral titer assays further showed that progeny virion release was not affected by CD81 blockade (Fig. 4N). These data indicate that CD81 acts mainly at the entry stage of PRRSV infection, rather than during viral attachment, genome replication, or virion release.

### The C-terminal transmembrane domain of CD81 is required for GP5 interaction and PRRSV replication

**Fig 5:**
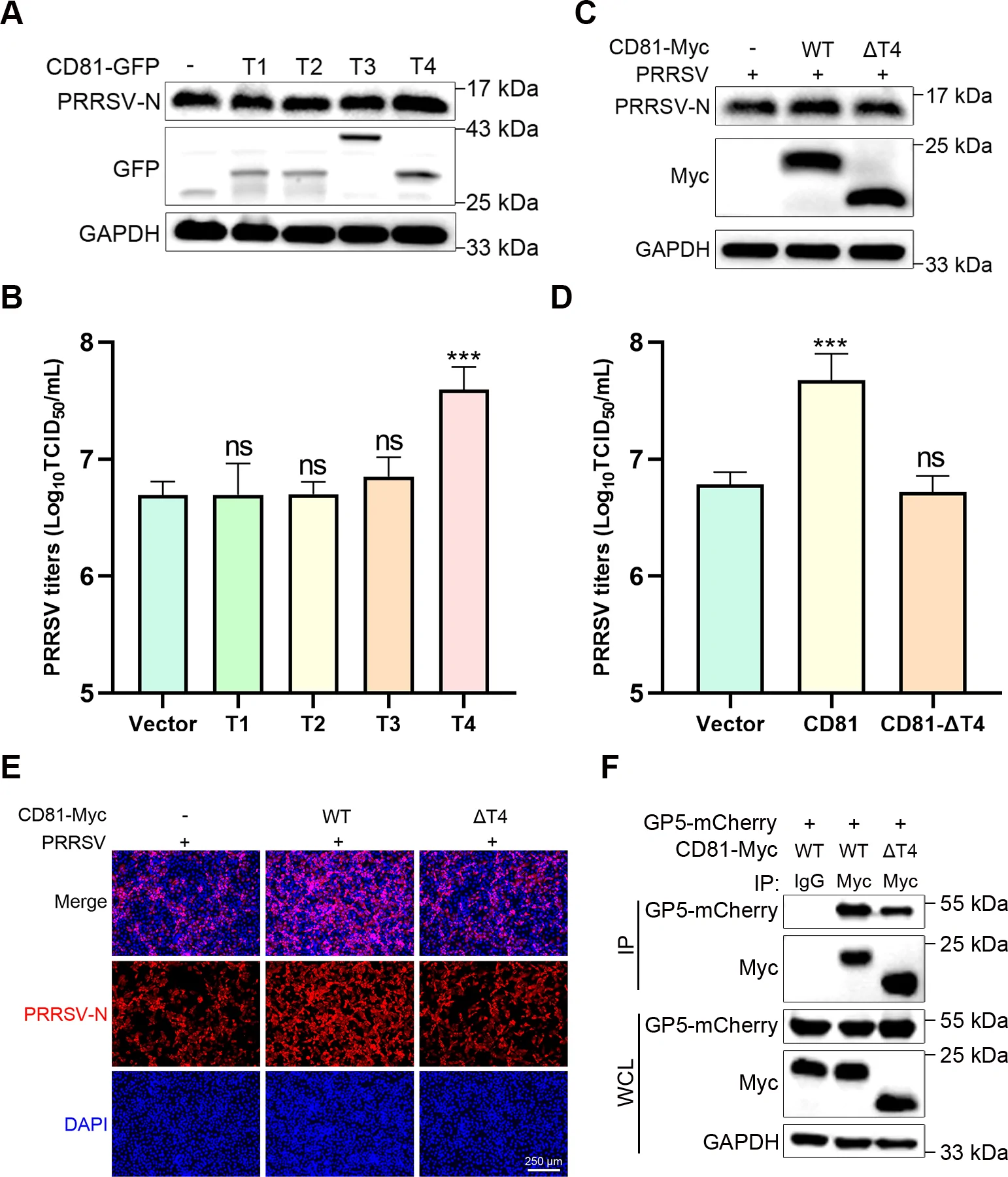
The C-terminal transmembrane domain of CD81 is critical for both GP5 interaction and viral replication**.** (**A and B**) Marc-145 cells were transfected with CD81-GFP truncations for 24 h and infected with PRRSV CH-1a (MOI 0.5) for 24 h. Viral N protein expression was analyzed by western blot (**A**), and virus titers in culture supernatants were determined using the TCID₅₀ assay (**B**). (**C–E**) Marc-145 cells were transfected with CD81 or CD81-ΔT4 for 24 h and infected with PRRSV CH-1a (MOI 0.5) for 24 h. Viral N protein levels were assessed by western blot (**C**), PRRSV titers in supernatants were measured by TCID₅₀ assay (**D**), and viral N protein fluorescence intensity was quantified by IFA (**E**). (**F**) HEK-293T cells were co-transfected with GP5-mCherry and either CD81-Myc or CD81-ΔT4, followed by Co-IP analysis. Data are shown as mean ± SEM (*n* = 3). Statistical significance was determined using one-way ANOVA. *P < 0.05, **P < 0.01, ***P < 0.001; ns, not significant.

To identify the CD81 region responsible for supporting PRRSV replication, a series of CD81 truncation mutants was examined. Among these mutants, only overexpression of the C-terminal transmembrane domain, T4 (aa 202–236), increased viral replication, as indicated by elevated N protein expression and higher progeny virus titers. Other truncated fragments did not produce a comparable effect (Fig. 5A and B). To further assess the requirement for this region, a CD81 mutant lacking T4 (CD81-ΔT4) was generated. Deletion of T4 abolished the proviral effect of CD81, with viral protein levels and virus titers returning to levels similar to those observed in the control group (Fig. 5C through E). Co-IP analysis showed that the ΔT4 mutation markedly weakened the interaction between CD81 and the viral glycoprotein GP5 (Fig. 5F). These results indicate that the C-terminal transmembrane domain of CD81 is required for its association with GP5 and for its ability to promote PRRSV replication.

### CD81 undergoes degradation via the autophagy pathway during PRRSV infection

**Fig 6:**
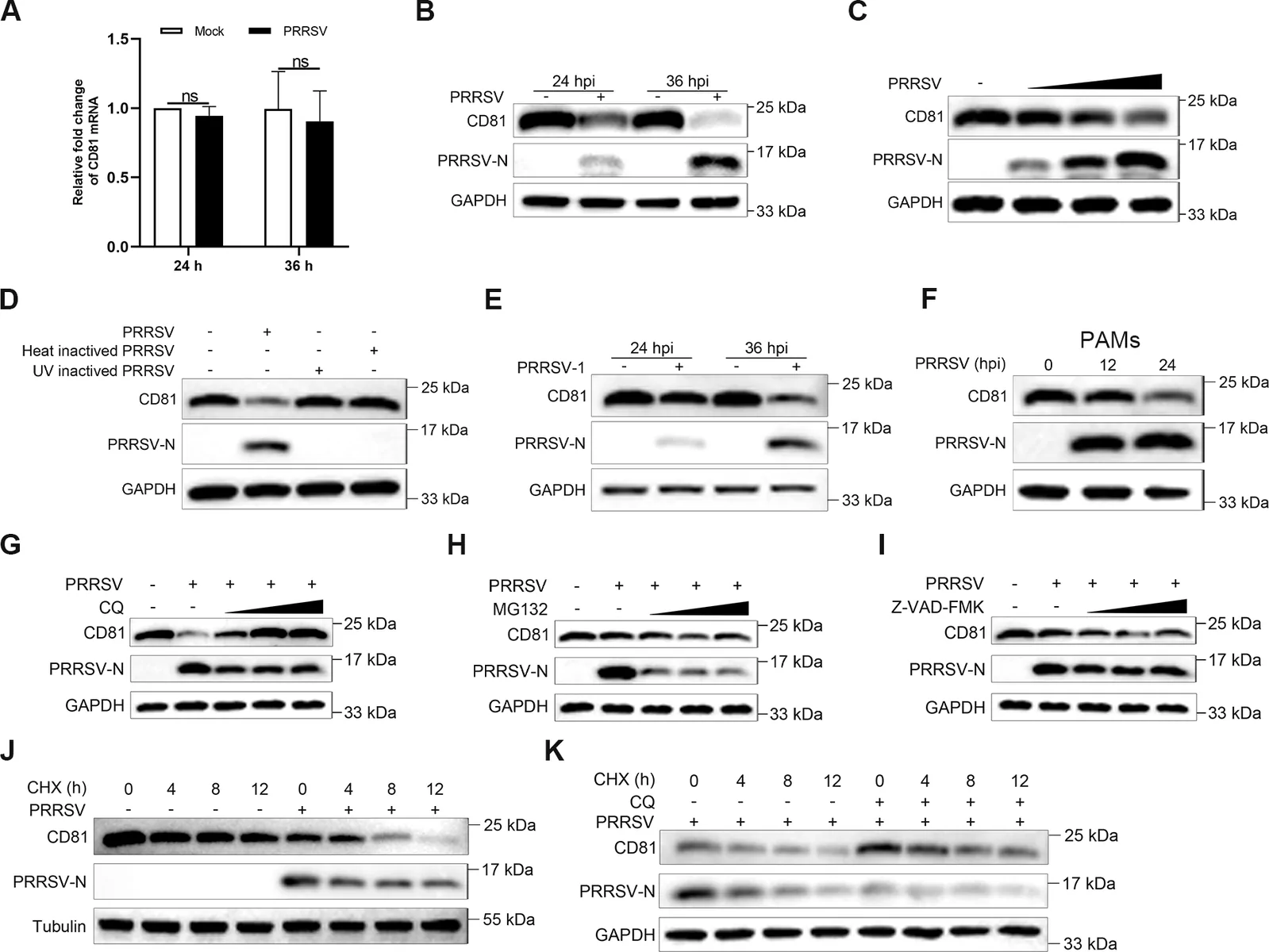
CD81 is degraded via the autophagy pathway during PRRSV infection. (**A and B**) Marc-145 cells were mock-infected or infected with PRRSV CH-1a (MOI 0.5) and harvested at the indicated time points, followed by RT-qPCR and western blot analysis. (**C**) Marc-145 cells were infected with PRRSV CH-1a at increasing MOIs (0, 0.05, 0.5, 5) for 24 h, followed by western blot analysis. (**D**) Marc-145 cells were mock-infected or infected with live, UV-inactivated, or heat-inactivated PRRSV CH-1a (MOI 0.5) for 36 h and analyzed for CD81 by western blot. (**E**) Marc-145 cells were mock-infected or infected with PRRSV-1 (MOI 0.5) for 24 or 36 h, followed by western blot analysis. (**F**) PAMs were mock-infected or infected with PRRSV JXA1 (MOI 0.5) for 12 or 24 h, followed by western blot analysis. (**G–I**) Marc-145 cells infected with PRRSV CH-1a (MOI 0.5) for 24 h were treated with the indicated concentrations of CQ (0, 12.5, 25, or 50 μM), MG132 (0, 2.5, 5, or 10 μM), or Z-VAD-FMK (0, 5, 10, or 20 μM) for the last 12 h, followed by western blot analysis. (**J**) Marc-145 cells mock-infected or infected with PRRSV CH-1a (MOI 0.5) for 24 h were treated with CHX (50 μg/mL) and harvested at the indicated time points for western blot analysis. (**K**) Marc-145 cells infected with PRRSV CH-1a (MOI 0.5) for 24 h were treated with CHX (50 μg/mL) in the presence or absence of CQ (50 μM) and harvested at the indicated time points for western blot analysis. Data are shown as mean ± SEM (*n* = 3). Statistical significance was determined using Student’s *t*-test. *P < 0.05, **P < 0.01, ***P < 0.001; ns, not significant.

To investigate whether PRRSV manipulates CD81 expression to facilitate infection, we first monitored CD81 dynamics in Marc-145 cells. Infection significantly reduced CD81 protein levels in a time- and dose-dependent manner without affecting its mRNA (Fig. 6A through C). This downregulation required infectious virions, as UV- or heat-inactivated virus had no effect (Fig. 6D), and was conserved across the PRRSV-1 and PAMs (Fig. 6E and F). To delineate the degradation route, we employed pathway-specific inhibitors. Notably, CD81 loss was rescued exclusively by the autophagy–lysosome inhibitor CQ, but not by proteasome (MG132) or apoptosis (Z-VAD-FMK) inhibitors (Fig. 6G through I). Consistently, CHX-chase assays showed that PRRSV infection accelerated CD81 degradation, and this acceleration was blocked by CQ cotreatment (Fig. 6J and K). Together, these results establish that the host counteracts PRRSV through an autophagy-dependent mechanism that actively degrades CD81, thereby limiting viral propagation.

### NDP52 regulates CD81 stability by modulating autophagy

**Fig 7:**
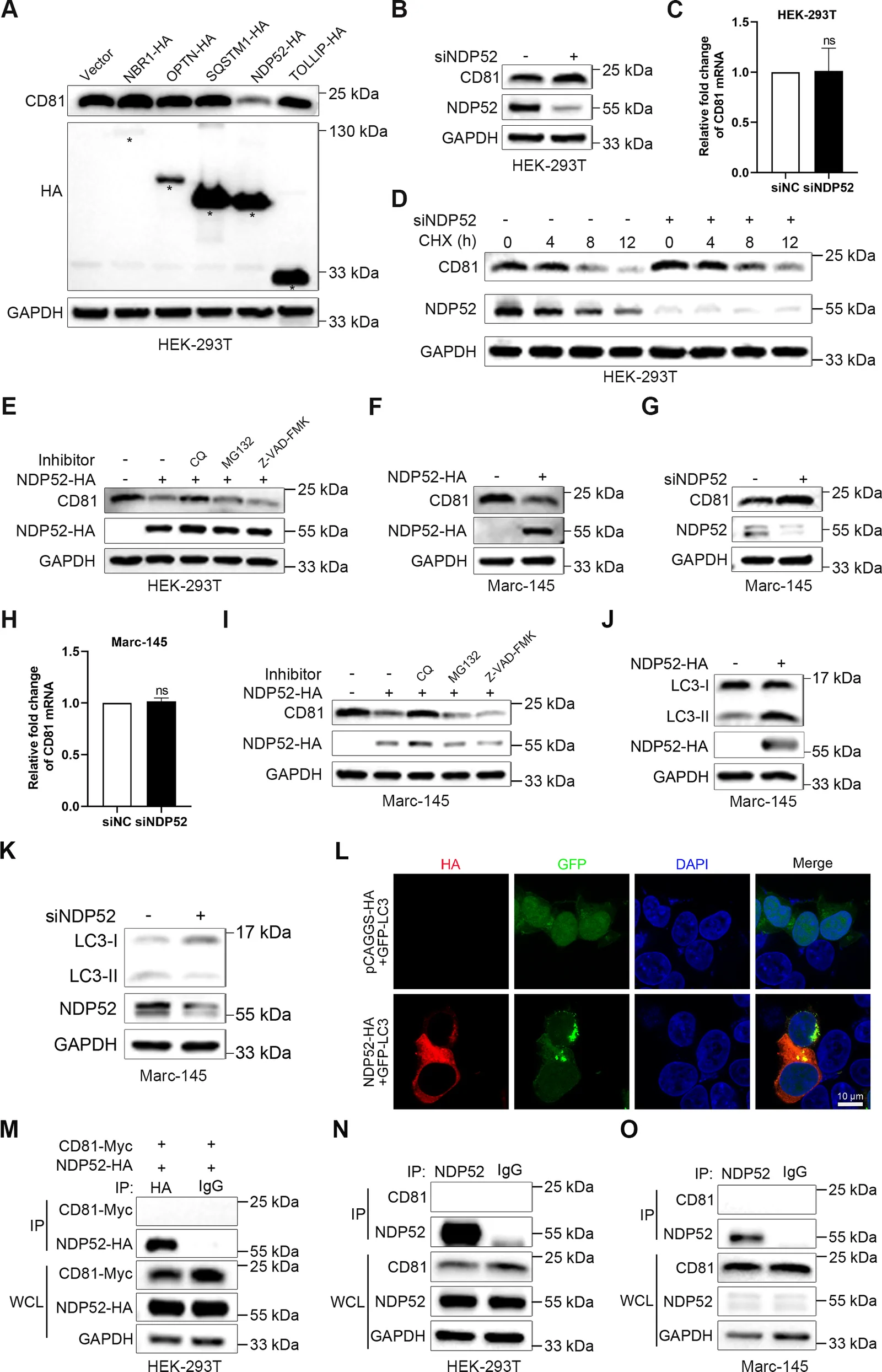
CD81 stability is regulated by NDP52 via its modulation of autophagy**.** (**A**) HEK-293T cells were transfected with plasmids encoding NBR1, OPTN, SQSTM1, NDP52, TOLLIP, or EV for 24 h and analyzed by western blot. (**B and C**) HEK-293T cells were transfected with siNC or siNDP52 for 24 h and analyzed by western blot (**B**) and RT-qPCR (**C**). (**D**) HEK-293T cells transfected with siNC or siNDP52 for 24 h were treated with CHX (50 μg/mL) and harvested at the indicated time points for western blot. (**E**) HEK-293T cells transfected with NDP52 or EV for 24 h were treated with CQ (50 μM), MG132 (10 μM), or Z-VAD-FMK (20 μM) for 12 h and analyzed by western blot. (**F**) Marc-145 cells transfected with NDP52 or EV for 24 h were analyzed by western blot. (**G and H**) Marc-145 cells were transfected with siNC or siNDP52 for 24 h and analyzed by western blot (**G**) and RT-qPCR (**H**). (**I**) Marc-145 cells transfected with NDP52 or EV for 24 h were treated with CQ (50 μM), MG132 (10 μM), or Z-VAD-FMK (20 μM) for 12 h and analyzed by western blot. (**J**) Marc-145 cells transfected with NDP52 or EV for 24 h were analyzed by western blot. (**K**) Marc-145 cells transfected with siNC or siNDP52 for 24 h were analyzed by western blot. (**L**) HEK-293T cells co-transfected with GFP-LC3 and NDP52 or EV were imaged by confocal microscopy to quantify LC3 puncta. (**M**) HEK-293T cells were co-transfected with CD81-Myc and NDP52-HA and subjected to Co-IP. (**N and O**) Lysates from HEK-293T (**N**) or Marc-145 (**O**) cells were immunoprecipitated with anti-NDP52 or control IgG beads and analyzed by western blot. Data are shown as mean ± SEM (*n* = 3). Statistical significance was determined using Student’s *t*-test. *P < 0.05, **P < 0.01, ***P < 0.001; ns, not significant.

Given that CD81 can be degraded by autophagy during PRRSV infection and that selective autophagy depends on specific receptors, especially considering that CD81 is primarily localized to the plasma membrane and thus unlikely to be targeted by receptors such as those involved in mitophagy or reticulophagy, we focused on investigating whether the autophagy receptors NBR1, OPTN, SQSTM1, NDP52, and TOLLIP mediate this process. Overexpression of NDP52, but not NBR1, OPTN, SQSTM1, or TOLLIP, effectively reduced endogenous CD81 levels in HEK-293T cells (Fig. 7A). Conversely, NDP52 knockdown increased CD81 protein abundance without affecting its mRNA and extended its half-life (Fig. 7B through D). The CD81 degradation driven by NDP52 overexpression was rescued by CQ (Fig. 7E), confirming an autophagic route. We validated this regulatory axis in the PRRSV-susceptible Marc-145 cell line: NDP52 overexpression promoted CD81 degradation, while its knockdown stabilized CD81, with CQ again rescuing the phenotype (Fig. 7F through I). These data indicate that NDP52 contributes to the autophagic-lysosomal degradation of CD81. We then asked whether this effect was associated with changes in autophagic activity. Overexpression of NDP52 increased autophagy, whereas NDP52 knockdown reduced autophagic activity, as reflected by changes in LC3-II levels and LC3 puncta formation (Fig. 7J through L). These results suggest that NDP52 supports autophagic flux under the experimental conditions tested. To assess whether NDP52 regulates CD81 through direct association, Co-IP assays were performed in both HEK-293T and Marc-145 cells. Neither exogenous nor endogenous NDP52 showed detectable interaction with CD81 (Fig. 7M through O). Thus, NDP52 appears to affect CD81 stability indirectly, most likely by promoting autophagic activity rather than by binding CD81 directly.

### PRRSV sustains CD81 stability by promoting the autophagic degradation of NDP52

**Fig 8:**
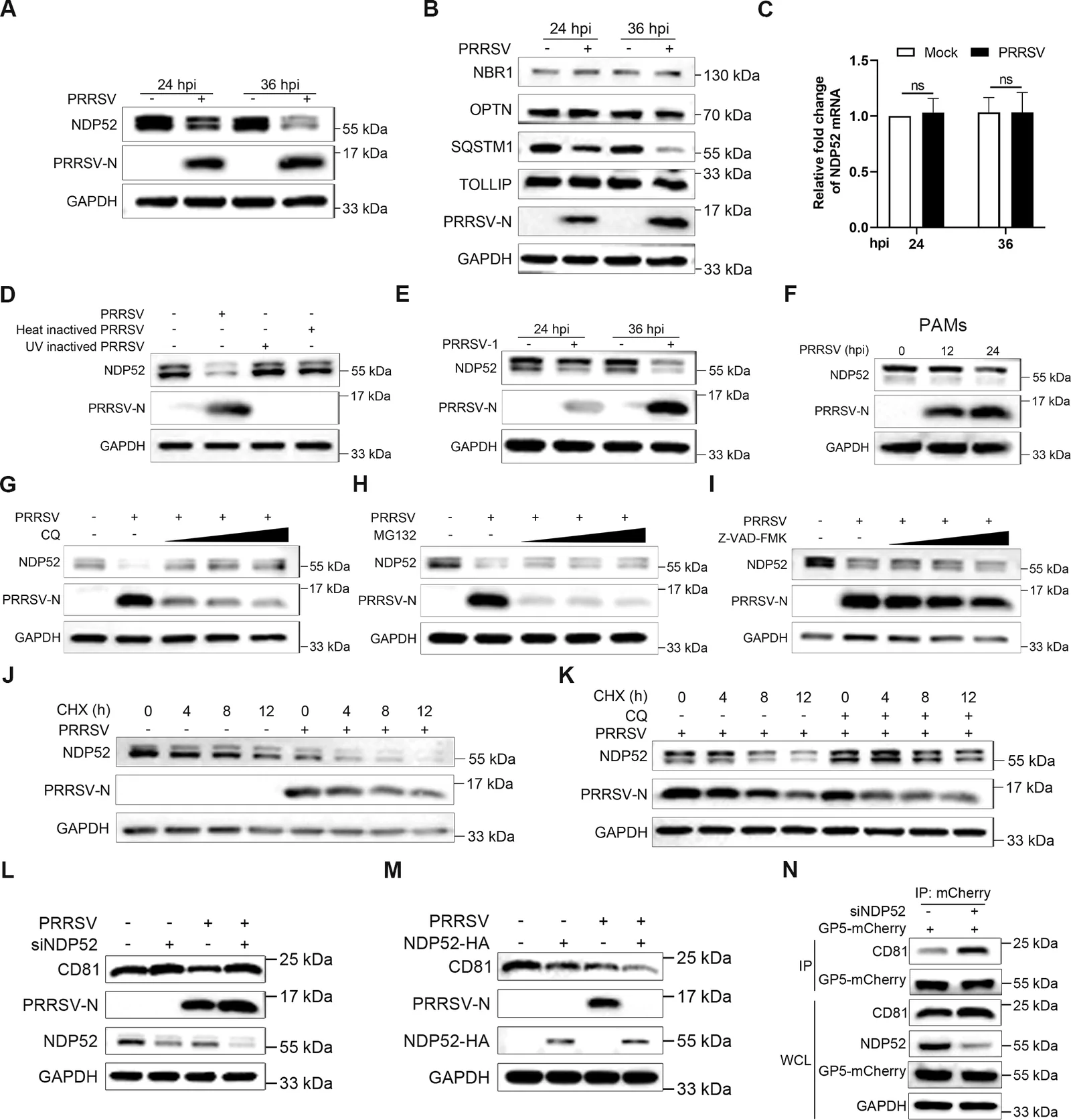
PRRSV targets NDP52 for autophagic degradation to maintain CD81 stability. (**A–C**) Marc-145 cells were mock-infected or infected with PRRSV CH-1a (MOI 0.5). At the indicated time points, cells were harvested for western blot (**A and B**) and RT-qPCR (**C**) analysis. (**D**) Marc-145 cells were mock-infected or infected with live, UV-inactivated, or heat-inactivated PRRSV CH-1a (MOI 0.5) for 36 h and analyzed for NDP52 by western blot. (**E**) Marc-145 cells were mock-infected or infected with PRRSV-1 (MOI 0.5) for 24 or 36 h and analyzed by western blot. (**F**) PAMs were mock-infected or infected with PRRSV JXA1 (MOI 0.5) for 12 or 24 h, followed by western blot analysis. (**G–I**) Marc-145 cells infected with PRRSV CH-1a (MOI 0.5) for 24 h were treated during the last 12 h of infection with increasing concentrations of CQ (0, 12.5, 25, and 50 μM), MG132 (0, 2.5, 5, and 10 μM), or Z-VAD-FMK (0, 5, 10, and 20 μM), followed by western blot analysis. (**J**) Marc-145 cells mock-infected or infected with PRRSV CH-1a (MOI 0.5) for 24 h were treated with CHX (50 μg/mL) and harvested at the indicated time points for western blot analysis. (**K**) Marc-145 cells infected with PRRSV CH-1a (MOI 0.5) for 24 h were treated with CHX (50 μg/mL) with or without CQ (50 μM) and harvested at the indicated time points for western blot analysis. (**L**) Marc-145 cells transfected with siNC or siNDP52 for 24 h were mock-infected or infected with PRRSV CH-1a (MOI 0.5) for a further 24 h and analyzed by western blot. (**M**) Marc-145 cells were transfected with NDP52 or EV for 24 h, then mock-infected or infected with PRRSV CH-1a (MOI 0.5) for 24 h, and analyzed by western blot. (**N**) HEK-293T cells were transfected with siNC or siNDP52 for 12 h, then with GP5-mCherry for 24 h, followed by Co-IP. Data are shown as mean ± SEM (*n* = 3). Statistical significance was determined using Student’s *t*-test. *P < 0.05, **P < 0.01, ***P < 0.001; ns, not significant.

Given the role of NDP52 in promoting CD81 degradation, we hypothesized that PRRSV might counteract this host defense by modulating NDP52. Indeed, PRRSV infection led to a time-dependent reduction in NDP52 protein levels (Fig. 8A). To investigate whether this downregulation is specific, we also assessed the expression of four other autophagy receptors during infection. Notably, SQSTM1 was similarly downregulated, whereas NBR1, OPTN, and TOLLIP remained largely unchanged, indicating that PRRSV differentially regulates autophagy receptor expression, with selective effects on NDP52 and SQSTM1 (Fig. 8B). To determine whether the reduction in NDP52 occurs at the transcriptional level, we measured its mRNA expression and found it unchanged, suggesting that PRRSV may regulate NDP52 through post-translational mechanisms (Fig. 8C). Importantly, heat-inactivated or UV-inactivated PRRSV failed to reduce NDP52 levels, indicating that active viral replication is required for this effect (Fig. 8D). This effect was conserved, as it was also observed with PRRSV-1 infection and in PAMs (Fig. 8E and F). To delineate the degradation pathway, we employed specific inhibitors. NDP52 loss was blocked by the autophagy inhibitor CQ, but not by the proteasome inhibitor MG132 or the apoptosis inhibitor Z-VAD-FMK (Fig. 8G through I). Accordingly, CHX experiments showed that NDP52 loss was accelerated during infection, and this acceleration was reversed by CQ cotreatment (Fig. 8J and K). We next assessed the functional impact of NDP52 modulation on CD81 stability and viral replication. NDP52 knockdown elevated basal CD81 levels, attenuated its infection-induced degradation, and enhanced viral replication (Fig. 8L). Conversely, NDP52 overexpression intensified CD81 degradation and suppressed viral replication (Fig. 8M). Further, NDP52 knockdown increased the interaction between CD81 and the PRRSV envelope protein GP5 (Fig. 8N). These findings support a model in which PRRSV promotes the autophagic degradation of NDP52 to stabilize CD81, thereby promoting CD81-GP5 binding and facilitating infection.

## DISCUSSION

Viral entry is the first step in productive infection and usually involves receptor engagement, membrane penetration or fusion, and subsequent release of the viral genome into the cytoplasm (32–36). In the case of PRRSV, entry is not mediated by a single receptor but depends on a coordinated set of host factors (37). Current evidence suggests that heparan sulfate proteoglycans contribute to initial attachment through interaction with the M/GP5 heterodimer, whereas CD163 engagement by the GP2a/GP3/GP4 complex is required for membrane fusion and viral RNA release (38–40). Given the central role of CD163 in PRRSV entry, it remains important to determine whether other CD proteins also participate in this process, particularly from the perspective of host-directed antiviral intervention.

To address this question, we performed a CRISPR knockout screen targeting CD proteins and identified CD81 as a pro-viral host factor. Y2H screening further indicated that CD81 interacts with GP5, and this interaction was validated by Co-IP and confocal microscopy. Domain-mapping analysis showed that GP5 ectodomain-containing regions interact with several transmembrane regions of CD81, rather than with the LEL, which is commonly used by viruses such as HCV and PCV2 (24, 28). This pattern suggests that PRRSV exploits CD81 through a mode distinct from the LEL-dependent mechanisms described for these viruses. Because CD81 has been implicated as a proviral cofactor in several viral infections (25, 28, 41), we further examined its role during PRRSV infection. In Marc-145 cells, loss- and gain-of-function analyses showed that CD81 knockdown restricted viral replication, whereas CD81 overexpression enhanced it, consistent with its supportive role in other viral systems (26, 42). Antibody-mediated blockade of CD81 also reduced PRRSV infection in both Marc-145 cells and PAMs, suggesting that the effect is not limited to a transformed cell line model. This observation is also in line with reports from other viral infections, including avian reovirus (ARV) (43).

Dissection of the PRRSV life cycle placed the function of CD81 mainly at the internalization stage, which is consistent with the known role of CD81 as an entry-associated cofactor for HCV, PCV2, and EAV (26, 28, 44–46). Truncation analysis further identified the C-terminal transmembrane domain of CD81 as the region required for its proviral effect, again differing from the LEL-dependent use of CD81 by HCV and PCV2 (24, 28). Given the importance of GP5 in PRRSV entry (47), we examined whether this domain was involved in GP5 binding. Deletion of the C-terminal transmembrane domain weakened the CD81-GP5 interaction and abolished the ability of CD81 to promote PRRSV replication. These findings indicate that the C-terminal transmembrane region of CD81 contributes to GP5 association and supports efficient PRRSV infection. Notably, although GP5 is known to form heterodimers with M protein on the virion surface, a complex essential for receptor attachment (48), our Y2H assays confirmed a direct interaction between GP5 and CD81, indicating that the GP5-M heterodimer does not preclude direct GP5-CD81 binding. On the basis of these observations, we propose a two-step model in which the GP5-M heterodimer primarily promotes viral attachment, while a subsequent direct GP5-CD81 interaction drives viral internalization. Whether the M protein cooperatively participates in the CD81-GP5 interaction and thereby influences internalization remains an open question for future investigation.

Although CD81 acts as a proviral factor, PRRSV infection also induced its autophagic degradation. This response may represent a host restriction mechanism that reduces the availability of CD81 and limits viral spread. Such post-translational regulation differs from the transcriptional downregulation of CD81 reported during HCV infection (49). Notably, although autophagy is generally thought to favor PRRSV replication, the degradation of CD81 via this pathway represents a host-directed antiviral response, highlighting the dual nature of autophagy in the host-virus arms race.

By screening selective autophagy receptors (50, 51), we identified NDP52 as an important mediator of CD81 turnover: NDP52 overexpression promoted CD81 degradation, whereas NDP52 knockdown stabilized CD81. Mechanistically, NDP52 did not directly bind CD81 but instead modulated autophagic activity, a finding consistent with its reported role in the autophagic degradation of EMCV proteins (52). We then examined whether PRRSV could counteract this restriction pathway. PRRSV infection promoted autophagic degradation of NDP52, thereby stabilizing CD81 and favoring infection. This strategy resembles that used by EMCV, which degrades NDP52 to evade autophagic clearance (52). In agreement with this model, NDP52 knockdown enhanced the CD81-GP5 interaction and facilitated PRRSV infection. Importantly, although PRRSV-mediated NDP52 degradation partially counteracted CD81 downregulation, the host-induced CD81 loss remained dominant, leading to an overall net reduction in CD81 levels during infection. Thus, this viral countermeasure represents a fine-tuning mechanism that alleviates, but does not fully reverse, the host restriction.

**Fig 9:**
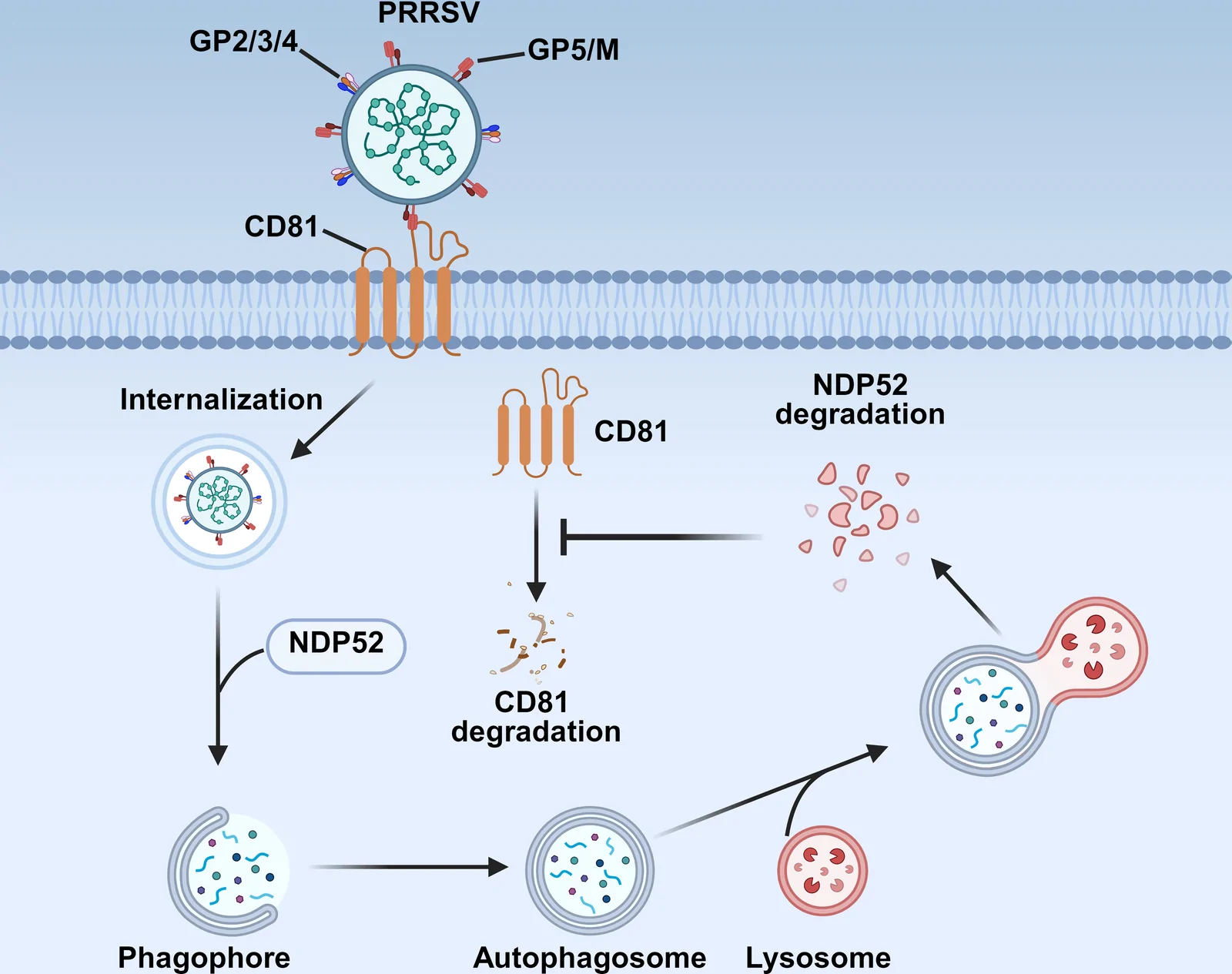
Schematic model of the host-virus interplay centered on CD81 during PRRSV infection. Upon infection, PRRSV engages the host membrane protein CD81 via its glycoprotein GP5 to promote viral entry and replication. In defense, the host targets CD81 for autophagic degradation to restrict infection. PRRSV counters this restriction by promoting degradation of the autophagy receptor NDP52, thereby stabilizing CD81 and creating conditions that favor viral propagation.

In summary, this study identifies a CD81-centered host-virus regulatory axis during PRRSV infection (Fig. 9). PRRSV uses the CD81-GP5 interaction to support viral entry, whereas host cells can restrict this process by promoting autophagic degradation of CD81. PRRSV, in turn, counteracts this restriction by inducing autophagic degradation of NDP52, leading to CD81 stabilization and enhanced infection. These findings elucidate a key layer of PRRSV pathogenesis and establish CD81 as a mechanistically validated target for novel antiviral strategies.

## MATERIALS AND METHODS

### Cells and viruses

Human embryonic kidney (HEK)-293T, 3D4/21, and Marc-145 cells were cultured in Dulbecco’s modified Eagle’s medium (DMEM; Bio-Channel) supplemented with 10% fetal bovine serum (FBS; Bio-Channel). PAMs were isolated from 4-week-old PRRSV-negative piglets using established protocols and maintained in RPMI 1640 medium (Gibco) containing 10% FBS. All cells were incubated at 37 °C under 5% CO₂. The following PRRSV strains were used in this study: PRRSV-2 strains JXA1 (GenBank: FJ548855.1) and CH-1a (AY032626.1), as well as the PRRSV-1 strain. The PRRSV-2 strain JXA1 is sensitive to PAMs and was used in PAM-related experiments. Viral stocks were propagated and titrated in Marc-145 cells.

### Antibodies and reagents

Anti-PRRSV N monoclonal antibody (JN0401) was purchased from MEDIAN Diagnostics (Korea); anti-PRRSV nsp1α polyclonal antibody (GTX133695) from GeneTex; anti-GAPDH (ab9485) from Abcam; mCherry polyclonal antibody (26765-1-AP), mCherry monoclonal antibody (68088-1-Ig), Myc monoclonal antibody (60003-2-Ig), Myc polyclonal antibody (16286-1-AP), NBR1 polyclonal antibody (16004-1-AP), OPTN polyclonal antibody (10837-1-AP), SQSTM1 polyclonal antibody (18420-1-AP), NDP52 polyclonal antibody (12229-1-AP), TOLLIP polyclonal antibody (11315-1-AP), LC3 polyclonal antibody (14600-1-AP), and HRP-conjugated anti-mouse (SA00001-1) and anti-rabbit (SA00001-2) antibodies from Proteintech Group; Alexa Fluor 488-conjugated goat anti-rabbit (4412) or anti-mouse (4408) and Alexa Fluor 555-conjugated goat anti-rabbit (4413) or anti-mouse (4409) antibodies from Cell Signaling Technology; anti-CD81 (sc-166029) and normal mouse IgG (sc-2025) from Santa Cruz Biotechnology; Protein A/G Magnetic Beads (B23202) from Selleck Chemicals; polyethylenimine (PEI; G1802) from Servicebio; 4′,6-diamidino-2-phenylindole (DAPI; C0065) from Solarbio; and NanoTrans Transfection Reagent 3000 (CT0006) from Shanghai Cytochiotechnology Co., Ltd. Other chemicals, including Cycloheximide (CHX; HY-12320), MG132 (HY-13259), chloroquine (CQ; HY-17589A), Z-VAD-FMK (HY-164388), and ER 50891(HY-108531), were purchased from MedChemExpress.

### Design and functional screening of a CRISPR sgRNA library targeting CD proteins

A CRISPR sgRNA library was designed to target CD proteins, with five distinct sgRNAs per gene, alongside two sets of control sequences. To minimize the enrichment of cells with multiple concomitant gene knockouts, the lentiviral-packaged sgRNA library was transduced into Marc-145-Cas9 cells at a low multiplicity of infection (MOI) of 0.3 to ensure single-copy integration per cell. Transduced cells were selected with puromycin (1 μg/mL) and blasticidin (5 μg/mL) for 14 days. Following selection, next-generation sequencing (NGS) of the integrated sgRNA cassette confirmed that >95% of the designed sgRNAs were retained in the selected cell pool. The engineered cells were then infected with PRRSV at a low MOI for 36 h. Surviving cells were expanded and subjected to two additional rounds of infection at progressively higher MOIs. This stepwise increase in viral challenge pressure effectively enriches for cells in which critical virus-dependent host factors have been successfully knocked out; only such cells can survive and expand under escalating viral pressure. After the final cycle, genomic DNA was extracted from the surviving cell pool, and the sgRNA regions were PCR-amplified and subjected to high-throughput sequencing. By comparing sgRNA abundances in the selected surviving population versus the original library, we identified significantly enriched sgRNAs. Host genes corresponding to highly enriched sgRNAs were considered critical candidate genes involved in resistance to PRRSV infection.

### Y2H screening for GP5-interacting proteins

The Y2H screening was performed as previously described (3). Briefly, lung tissues from PRRSV-infected and uninfected piglets were collected and pooled for cDNA library construction. The viral GP5 was cloned to generate the GP5-pBT3-SUC bait plasmid. To exclude false positives and assess potential bait-induced toxicity in yeast cells, a preliminary screening test was performed to evaluate bait self-activation. The cDNA library was constructed in the pPR3-N-DEST vector to generate prey plasmids. These prey plasmids were co-transformed with the GP5-pBT3-SUC bait plasmid into the yeast strain NMY51. Blue-positive colonies were selected and plated on SD/-Leu/-Trp/-His/-Ade/X-α-gal/AbA (QDO/X/A) plates, followed by sequencing and analysis. Positive (pTSU2-APP/pNubG-Fe65) and negative (GP5-pBT3-SUC/pPR3-N) control transformations were included in parallel.

### Plasmid construction and cell transfection

The full-length coding sequence of CD81 (GenBank accession no. XM_008007267) was amplified and cloned into the pcDNA3.1-Myc vector to generate the CD81-Myc expression plasmid. Truncated CD81 variants were PCR-amplified using CD81-Myc as a template and subcloned into pcDNA3.1-GFP. HA-tagged NBR1, OPTN, SQSTM1, NDP52, and TOLLIP were individually cloned into pCAGGS-HA. The gene encoding viral GP5 was amplified from the PRRSV CH-1a strain and cloned into pmCherry-N1 to produce GP5-mCherry, which subsequently served as the template for generating mCherry-tagged GP5 truncations. All plasmid constructs were confirmed by DNA sequencing. For transient transfection, HEK-293T or Marc-145 cells were seeded in culture plates and transfected at approximately 80% confluence using PEI or NanoTrans Transfection Reagent 3000 according to the manufacturer’s protocols.

### Reverse transcription-quantitative PCR (RT-qPCR)

Total RNA was extracted using TRIeasyReagent (YEASEN, 10606ES60). cDNA was synthesized from the purified RNA using ABScript Neo RT Master Mix for qPCR with gDNA Remover (ABclonal, RK20433). Quantitative PCR was performed on a QuantStudio 3 Real-Time PCR System (Thermo Fisher Scientific) with BrightCycle Blue Universal SYBR Green qPCR Mix with UDG (ABclonal, RK21220). Primer sequences are listed in Table S1. GAPDH (for Marc-145 cells) and HPRT1 (for PAMs) served as endogenous controls for normalization. Relative expression levels were calculated using the 2^−ΔΔCT^ method.

### Co-IP assay

HEK-293T cells were co-transfected with the indicated plasmids for 24 h. Cells were then collected, lysed on ice for 30 min using IP lysis buffer (Beyotime, P0037) supplemented with 1 mM PMSF (Solarbio, P0100). After centrifugation at 12,000 × *g* for 10 min at 4°C, the supernatants were incubated with magnetic beads pre-conjugated with the relevant antibodies for 2 h at room temperature or overnight at 4°C. The bead complexes were then washed three times with wash buffer, transferred to a new tube, and washed an additional three times. Finally, the immunoprecipitated proteins were eluted and prepared for western blot analysis.

### Western blot analysis

Following lysis with a buffer (Beyotime, P0013B) containing protease inhibitors (Solarbio, P0100), equal amounts of protein lysates were subjected to SDS-PAGE. The resolved proteins were then transferred onto PVDF membranes (Merck Millipore, ISEQ00010). After blocking with 5% nonfat milk (Beyotime, P0216) for 2 h at room temperature, the membranes were probed with primary antibodies and corresponding HRP-conjugated secondary antibodies. Signal detection was performed using an ECL kit (MeilunBio, MA0186-3).

### Indirect immunofluorescence assay (IFA)

The samples were fixed with 4% paraformaldehyde (Beyotime, P0099) for 15 min at room temperature and permeabilized with 0.5% Triton X-100 (Beyotime, P0096) for 5 min. After blocking with 1% bovine serum albumin (BSA; Beyotime, ST2249) for 1 h at room temperature, the cells were incubated with the indicated primary antibodies overnight at 4°C. Following three washes with PBS, the samples were exposed to secondary antibodies for 1 h at 37°C. Nuclei were visualized by counterstaining with DAPI for 5 min. Imaging was performed using either an inverted fluorescence microscope (Nikon ECLIPSE Ti2) or a confocal laser scanning microscope (Leica SP8).

### RNA interference (RNAi)

Small interfering RNAs (siRNAs) targeting CD81 and NDP52 were designed and synthesized by GenePharma (Shanghai, China) and IGE Biotech (Guangzhou, China), respectively. Marc-145 or HEK-293T cells cultured in 12-well plates were transfected with CD81-specific siRNA, NDP52-specific siRNA, or a non-targeting control siRNA using NanoTransTransfection Reagent 3000, following the manufacturer’s protocol. At 24 h post-transfection, cells were collected, and the knockdown efficiency was evaluated by RT-qPCR or western blot. The sequences of all siRNAs used in this study are listed in Table S2.

### Blocking experiment with anti-CD81 antibody

Cells were seeded in 12-well plates and cultured until reaching 70–80% confluency. The culture medium was aspirated, and the monolayers were washed three times with sterile phosphate-buffered saline (PBS). Subsequently, the cells were treated with varying final concentrations of anti-CD81 antibody or an isotype control antibody for 2 h at 37°C. Following pretreatment, the cells were infected with PRRSV at an MOI of 0.5. Finally, cell samples were collected at 24 h post-infection for further analysis.

### Viral titers

The viral titers present in cell culture supernatants were determined by measuring the 50% tissue culture infectious dose (TCID_50_) per milliliter. Briefly, Marc-145 cells were seeded as monolayers in 96-well plates and inoculated with serial tenfold dilutions of the supernatant samples. After incubation for 72 h, the cytopathic effect (CPE) was examined and recorded. The TCID_50_ values were subsequently calculated using the Reed-Muench method.

### Statistical analysis

All statistical analyses were performed using GraphPad Prism software (version 8.0). Between-group comparisons were performed using Student’s *t*-test, while multi-group comparisons were conducted using one-way or two-way analysis of variance (ANOVA), as appropriate. All data are presented as mean ± standard deviation (SD) based on at least three independent biological replicates, unless otherwise stated. Statistical significance was set at *P* < 0.05. Significance levels are denoted as follows: *P < 0.05, **P < 0.01, and ***P < 0.001; non-significant differences are indicated as “ns.”

## Data Availability

All data are available in the figures or can be provided upon request.

## SUPPLEMENTAL MATERIAL

Supplemental material jvi.01033-26-s0001.docx Figure S1 and Tables S1 and S2.

Files S1 and S2 jvi.01033-26-s0002.xlsx File 1, functional screening of a CRISPR sgRNA library targeting CD-family proteins; File 2, yeast two‑hybrid screening for PRRSV GP5‑interacting proteins.

ASM does not own the copyrights to Supplemental Material that may be linked to, or accessed through, an article. The authors have granted ASM a non-exclusive, world-wide license to publish the Supplemental Material files. Please contact the corresponding author directly for reuse.
