# Supplementary material for: CRISPR and yeast two-hybrid screens identify CD81 as a proviral host factor for PRRSV
Source: J Virol. 2026 Sep 14;100(10):e01033-26. doi: 10.1128/jvi.01033-26 (PMC13712945; doi:10.1128/jvi.01033-26)
Supplement: Supplementary file 1 [file jvi.01033-26-s0001.docx]

Supplementary Materials for

**CRISPR and yeast two-hybrid screens identify CD81 as a proviral host factor for PRRSV**

Yongjie Chen^1^, Zhan He^1^, Yanfei Pan^1^, Yunyan Luo^1^, Baoying Huang^1^, Yi Zhang^1^, Guohao Li^1^, Chunhe Guo ^1^*

*Corresponding author: Dr. Chunhe Guo; E-mail address: guochunh@mail.sysu.edu.cn;

This file contains Figure S1 and Tables S1 and S2.

**Supplementary figure and figure legend**

**Figure S1**

**Figure S1. Anti-CD81 antibody inhibits PRRSV infection in Marc-145 cells.** (A) Marc-145 cells were treated with various concentrations of anti-CD81 or anti‑IgG antibodies for 24 h, followed by a CCK-8 assay. (B-F) Marc-145 cells were pre-incubated with specific antibodies or isotype-control IgG for 2 h at 37°C before infection with PRRSV CH-1a (MOI 0.5). At 24 h post-infection, cells were analyzed by RT-qPCR (B and C), western blot (D), IFA (E), and TCID₅₀ (F) assay.

**Supplementary tables**

**Table S1. Primer sequences used in this study.**

| Primer name^a^ | Sequence (5’-3’) |
| --- | --- |
| PRRSV-N-F | AAAACCAGTCCAGAGGCAAG |
| PRRSV-N-R | CGGATCAGACGCACAGTATG |
| PRRSV-nsp1α-F  PRRSV-nsp1α-R | ACACGATGCCTCAGTGCA  CTCTTCGGGCCTGTAGAAT |
| mGAPDH-F | TGACAACAGCCTCAAGATCG |
| mGAPDH-R | GTCTTCTGGGTGGCAGTGAT |
| pHPRT1-F | TGGAAAGAATGTCTTGATTGTTGAAG |
| pHPRT1-R | ATCTTTGGATTATGCTGCTTGACC |
| hGAPDH-F | AAGGTCGGAGTCAACGG |
| hGAPDH-R  CD81-F  CD81-R | GGAAGATGGTGATGGGATT  TACATCCTCATTGCCGTGGG CATCCTTGGCAATCTGGTCCT |
| mNDP52-F | GGGATGGAAGACAGTCCGTG |
| mNDP52-R | TGCAAAGCTCCTTGTTGTGC |

^a^The prefix letters in the primer names indicate the species of origin: "m" for monkey, "p" for pig, and "h" for human.

**Table S2. Sequences of siRNA targeting CD81 and NDP52 in this study.**

| siRNA | Direction | Sequence (5’-3’) |
| --- | --- | --- |
| siCD81-1 | sense | GCGCUGUGAUGAUGUUCGUTT |
| siCD81-1 | antisense | ACGAACAUCAUCACAGCGCTT |
| siCD81-2 | sense | GCUCAAGAACAAUCUGUGUTT |
| siCD81-2 | antisense | ACACAGAUUGUUCUUGAGCTT |
| siCD81-3 | sense | CCGGGAAGCUGUACCUCAUTT |
| siCD81-3  siNDP52  siNDP52 | antisense  sense  antisense | AUGAGGUACAGCUUCCCGGTT  GGAACUGAAAGUGAAAGAATT  UUCUUUCACUUUCAGUUCCTT |
| siNC | sense | UUCUCCGAACGUGUCACGUTT |
| siNC | antisense | ACGUGACACGUUCGGAGAATT |
